# Fucoidan and Cancer: A Multifunctional Molecule with Anti-Tumor Potential

**DOI:** 10.3390/md13042327

**Published:** 2015-04-14

**Authors:** Farzaneh Atashrazm, Ray M. Lowenthal, Gregory M. Woods, Adele F. Holloway, Joanne L. Dickinson

**Affiliations:** 1 Menzies Institute for Medical Research, University of Tasmania, Hobart, Tasmania 7000, Australia; E-Mails: Farzaneh.atashrazm@utas.edu.au (F.A.); R.M.Lowenthal@utas.edu.au (R.M.L.); G.M.Woods@utas.edu.au (G.M.W.); 2 School of Medicine, University of Tasmania, Hobart, Tasmania 7000, Australia; E-Mail: A.F.Holloway@utas.edu.au

**Keywords:** fucoidan, cancer, apoptosis, synergy

## Abstract

There is a wide variety of cancer types yet, all share some common cellular and molecular behaviors. Most of the chemotherapeutic agents used in cancer treatment are designed to target common deregulated mechanisms within cancer cells. Many healthy tissues are also affected by the cytotoxic effects of these chemical agents. Fucoidan, a natural component of brown seaweed, has anti-cancer activity against various cancer types by targeting key apoptotic molecules. It also has beneficial effects as it can protect against toxicity associated with chemotherapeutic agents and radiation. Thus the synergistic effect of fucoidan with current anti-cancer agents is of considerable interest. This review discusses the mechanisms by which fucoidan retards tumor development, eradicates tumor cells and synergizes with anti-cancer chemotherapeutic agents. Challenges to the development of fucoidan as an anti-cancer agent will also be discussed.

## 1. Introduction to Cancer

Cancers are multifactorial diseases of various etiologies. They arise largely as a result of acquired genetic changes that alter cell function leading neoplastic cells to gain survival or growth advantages [1]. For cancer cells to survive, the generation of new blood vessels (angiogenesis) is required. Cancer leads to death mostly through tumor cell spread to distal organs (metastasis). Various pathways are disrupted in tumor development, which result from unbalanced programmed cell death, disordered signaling pathways, angiogenesis and poor immune response against cancer. Most of the chemotherapeutic agents used in cancer treatment target these major deregulated pathways. Unfortunately, as many of these therapies cause severe side effects, the toxicities limit the dose and thus the efficacy of treatment. Therefore, there is strong interest in developing better-tolerated anti-cancer agents.

## 2. A Role for Natural Products for Cancer Treatment

Chemotherapy has been a cornerstone of the standard cancer treatment regimens since the 1960s. A variety of chemicals ranging from traditional agents such as methotrexate and folic acid analogues to novel chemicals such as anthracyclines have been used in cancer treatment [2]. Despite promising tumor growth-inhibitory effects in pre-clinical tests, many fail in clinical trials when adverse unexpected side effects are revealed. Traditionally anti-cancer chemotherapy targets rapidly dividing and proliferating cells. Therefore, normal cells which have high-proliferating potential are also affected.

Novel therapeutic agents are designed to target specific molecules (targeted therapy). However, these targeted therapies are not always completely free of side effects either. For instance, vemurafenib, a B-Raf enzyme inhibitor, is specific for oncogenic mutant V600E B-Raf positive melanoma cells. This drug was the first targeted molecular therapy, which was approved for use in advanced stages of melanoma. Although vemurafenib has shown significant beneficial anti-cancer effects, several studies have reported the rapid emergence of acquired resistance and adverse dermatological effects. It also stimulates B-Raf expression in V600E B-Raf negative patients promoting melanoma growth [3,4]. Monoclonal antibodies are another example of targeted therapy and are designed to specifically target the cancer antigens located on tumor cells. Monoclonal antibodies are generally safer than chemotherapy and the side effects caused by them include mild allergic reactions such as urticaria. But they can also cause severe reactions such as infusion reactions and serum sickness. As an example, rituximab (anti-CD20), which is widely used in treating B-cell lymphoma, generally causes only mild toxicities, however, reports have described occasional cases with severe complications such as anaphylactic reactions and myocardial infarction as well as high risk of tumor lysis syndrome in patients who have a high burden of tumor cells in their circulation [5].

Concerns over toxicity, tumor cell resistance and development of secondary cancers from chemotherapeutic chemicals have generated interest in exploiting natural products for cancer treatment. Flavopiridol is a flavonoid derived from the indigenous Indian plant *Dysoxylum binectariferum*, which inhibits cell cycle progression. It is the first cyclin-dependent kinase (CDK) inhibitor to be approved for use in clinical trials [6]. Natural products are also being tested as adjuvants for use in synergy with chemotherapeutic agents. For example those with immunomodulatory effects can reduce immune suppression and the associated increased risk of infection. In George *et al.* [7] study, *Indukantha Ghritha* (IG), a polyherbal preparation consisting of 17 plant components, was used as an adjuvant to cyclophosphamide cancer chemotherapy and shown to stimulate the hematopoietic system and induce leukopoiesis in tumor-bearing mice. When administrated in combination with cyclophosphamide, it reversed myelosuppression induced by cyclophosphamide suggesting its potential to minimize or reverse chemotherapy-induced leukopenia.

Polysaccharides include a large family of diverse biopolymers. They are constituted by monosaccharide residues linked together by *O*-glycosidic bonds that are found in natural and semi-synthetic structures [8]. Due to structural diversity, polysaccharides display the highest biological properties among macromolecules. Many natural polysaccharides obtained from natural sources such as plants and algae have anti-cancer properties. The multifunctional structure of natural polysaccharides also allows them to be used in conjugation with anti-cancer agents that lack physiochemical and biopharmaceutical properties [8,9].

## 3. Fucoidan

Fucoidan is a natural sulfated polysaccharide that exists mainly in the cell wall matrix of various species of brown seaweed such as mozuku, kombu, limumoui, bladderwrack and wakame [10]. Various forms of fucoidan have also been recognized in some marine invertebrates such as sea urchins [11] and sea cucumbers [12]. The brown seaweeds containing fucoidan are widely consumed as part of the normal diet in East Asia, particularly Japan, China and Korea.

### 3.1. Fucoidan’s Anti-Cancer Potential

The anti-cancer property of fucoidan has been demonstrated *in vivo* and *in vitro* in different types of cancers. Nevertheless, it has been rarely investigated for its anti-cancer properties in clinical trials. Fucoidan mediates its activity through various mechanisms such as induction of cell cycle arrest, apoptosis and immune system activation. Additional activities of fucoidan have been reported that may be linked to the observed anti-cancer properties and these include induction of inflammation through immune system, oxidative stress and stem cell mobilization. These activities have been reviewed by Kwak [13].

#### 3.1.1. Fucoidan and Cell Cycle

Fucoidan treatment results in sub G0/G1 cell accumulation (suggestive of dead cells/apoptotic cells) in a variety of cell types [14,15]. It can also induce cell cycle arrest in other phases; Riou *et al.* [16] and Mourea *et al.* [17] reported arrest in G1 phase in a chemo-resistant non-small-cell bronchopulmonary carcinoma line by fucoidan from *Ascophyllum nodosum* and *Bifurcaria bifurcate*, respectively.

In an investigation of the mechanism of the action, fucoidan demonstrated significant down regulation of cyclin D1, cyclin D2 and CDK4 in cancer cells [18,19,20]. The crude fucoidan from *Fucus vesiculosus* increased the level of p21/WAF1/CIP1 in PC3 cells and down-regulated E2F; a transcription factor that controls progression of cells from G1 to S phase [18].

**Table 1:** Effects of fucoidan on cell cycle and apoptosis molecules.

Ref	Cell Type	Fucoidan Source	Dose (µg/mL)	Effects on Cell Cycle	Effects on Apoptosis Pathways Extrinsic Intrinsic Common		
[15]	Human lymphoma HS-sultan cells	**F. vesiculosus**	100	↑ sub G0/G1	-	↓ MMP	Caspase 3 activation
				No G0/G1 or G2/M arrest			
[20]	HTLV-1 infected T-cell HUT-102- cells	*C. okamurans*	3000	G1 arrest	Apoptosis was reversed by caspase 8 inhibitor	Caspase 9 activation	Apoptosis was reversed by caspase 3 inhibitor
				↓ cyclin D2, c-myc		No changes in Bcl-2 and Bcl-XL	
				No changes in p21,p53		↓ survivin, cIAP-2	
[21]	Human hepatocellular carcinoma cells	*Okinawa mozuku*	22.5	↑ G2/M phase in HAK-1A, KYN-2, KYN-3 cell lines	-	No clear caspase 9 activation in HAK-1B cell line	No clear caspase 3 activation in HAK-1B cells
[22]	Human breast cancer MCF7 cells	Not mentioned	1000	↑ sub-G1 fraction	Caspase 8 activation	Caspase 9 activation	Caspase 7 activation
					Caspase inhibitors blocked apoptosis completely	↓ Bid, cytosolic Bax	PARP cleavage
						↑ whole lysate Bax, cytosolic cytochrome C	
[23]	Human acute leukemia NB4 and HL-60 cells	**F. vesiculosus**	150	↑ sub-G1 fraction	Caspase 8 activation	caspase 9 activation	PARP cleavage
						No changes in Bcl-2 or Bax	Caspase 3 activation
						↓ Mcl-1, ↑ cytochrome C	
[24]	Human colon cancer HT-29 and HCT116 cells	*F. vesiculosus*		-	Caspase 8 activation	Caspase 9 activation	PARP cleavage
					↑ Fas, DR5, TRAIL	↑ cytochrome C, Smac/Diablo, Bak, t-Bid	Caspase 3 and 7 activation
					No significant effects on FasL and DR4	No changes in Bcl-2, Bcl-xL, Bax, Bad, Bim, Bik	
						↓ XIAP, survivin	
[25]	Human lung cancer A549 cells	*U. pinnatifida*	50, 100, 200	↑ Sub-G1frction	-	Caspase-9 activation	↓ procaspase-3
						↓ Bcl-2, ↑ Bax	PARP cleavage
[14]	Human breast cancer MCF-7 cells	*Cladosiphon novae-caledoniae*	82, 410, 820	↑ Sub-G1	No changes in caspase-8	Mitochondrial dysfunction	No activation of PARP and caspase-7
				No significant changes in cell cycle distribution		AIF and cytochrome C release	
						No cleavage of caspase-9 and Bid.	All caspase inhibitors failed to attenuate FE-induced apoptosis
						↓ Bcl-2, Bcl-xl ,↑ Bax, Bad	
[26]	Hela cells	*Sargassum filipendula*	1500	-	-	No effect on caspase 9 activation	No effect on caspase 3 (Caspase independent)
						↑ cytosol AIF	
[19]	Human breast cancer MCF-7 cells	*F. vesiculosus*	400, 800, 1000	G1 phase arrest	Caspase-8 activation	↓ Bcl-2	Caspase-dependent pathway
				↑ Sub G0/G1 ↓ cyclin D1 and CDK-4 gene expression		↑ Bax	
						Release of cytochrome C and APAf-1	
[18]	Human prostate cancer PC-3 cells	*U. pinnatifida*	100	G0/G1 phase arrest	DR5, caspase-8 activation	↓ Bcl-2	Caspase-3 activation
				↓ E2F-1		↑ Bax,	PARP cleavage
				↑ p21Cip1/Waf		Caspase 9 activation	
[27]	Human Hepatocellular Carcinoma SMMC-7721 cells	*U. pinnatifida*	1000	Non-significant accumulation in S-phase	Caspase-8 activation	Caspase-9 activation	Caspase-3 activation
						MMP dissipation, Cytochrome C release	
						↓ Bcl-2, ↑ Bax	
						↓ *XIAP*, livin mRNA expression	
[28]	Human bladder carcinoma 5637 and T-24 cells	*F. vesiculosus*	100	↑ G1-phase, p21WAF1	-	-	-
				↓ Cyclin E, D1, DK2, CDK4			
				No change in p27KIP,p53			
				↑ p21WAF1 and CDK4 binding			

In a recent study, fucoidan down-regulated cyclin E, CDK2, CDK4 resulting in G0/G1 arrest in human bladder cancer 5637 cells. Furthermore, immunoprecipitation assays revealed a significant increase in the binding of p21/WAF1/CIP1 to CDK2 and CDK4 in cells treated with fucoidan, suggesting that the induced G0/G1 arrest is due to suppression of CDK activity following direct binding of this CDK inhibitor to CDKs 2 and 4 [28]. Table 1 summarizes findings of studies examining the effects of fucoidan on cell cycle.

#### 3.1.2. Fucoidan and the Apoptosis Pathway

Apoptosis characterized by cytoplasmic shrinkage and chromatin condensation facilitates the removal of cells without inducing inflammation [29]. Apoptosis occurs through either the extrinsic (cytoplasmic) pathway whereby death receptors trigger the apoptosis, or the intrinsic (mitochondrial) pathway in which changes in mitochondrial membrane potential (MMP) lead to cytochrome C release and death signal activation. Both pathways activate executive caspases that cleave regulatory and structural molecules [30]. Several studies examining a variety of cancers such as hematopoietic, lung, breast and colon cancers have shown that fucoidan-mediated cell death occurs through triggering apoptosis (Table 1) [14,22,24]. A very low dose of fucoidan from *F. vesiculosus* (20 µg/mL) activated common caspases 3 and 7 in human colon cancer cells [24], whereas it induced the same activity in T-cell leukemia at a much higher concentration (3 mg/mL) [20]. Caspase 8 and 9, two of the best characterized molecules of the extrinsic and intrinsic pathways respectively are activated by fucoidan [24]. Yamasaki-Miyamoto *et al.* showed that pre-treatment with caspase 8 inhibitor completely blocked fucoidan mediated apoptosis in MCF-7 breast cancer cell line [22]. In contrast, in Zhang *et al.* [14] study, the mediated apoptosis by fucoidan from *Cladosiphon okamuranus* in MCF-7 human breast cancer cell line was shown to be caspase independent. As cytochrome C and apoptosis inducing factor (AIF) increased in the cytosol, it was concluded that fucoidan performed its activity through mechanisms altering mitochondrial function.

Fucoidan also affects other components of extrinsic and intrinsic pathways. Analyzing the extrinsic pathway, 20 µg/mL crude fucoidan from *F. vesiculosus* increased the levels of the death receptors Fas, DR5 and TRAIL but not FasL and DR4 in human colon cancer cell lines [24]. Bcl-2 family members include anti-apoptotic, pro-apoptotic and regulatory proteins, which are mainly involved in the apoptosis intrinsic pathway. Contradictory results have been described in the expression of these regulatory molecules in response to fucoidan (Table 1). Treatment of MDA-MB231 breast cancer cells with 820 µg/mL of low molecular weight (LMW) fucoidan resulted in a significant decrease in anti-apoptotic proteins Bcl-2, Bcl-xl and Mcl-1 [31]. In contrast, no changes in expression of Bcl-2, Bcl-xl, Bad, Bim and Bik were observed in colon cancer cells when they were treated with 20 µg/mL fucoidan from *Fucus vesiculosus* [24]. Taken together, the results suggest that fucoidan may interact with several components of the apoptosis pathway.

#### 3.1.3. Fucoidan and Angiogenesis

Fucoidan inhibits the formation of new vessels by which tumor cells receive their oxygen and required nutrients. Fucoidan has been found to inhibit the binding of VEGF, a key angiogenesis promoting molecule, to its cell membrane receptor [32]. Xue *et al.* examined the anti-angiogenic properties of fucoidan in 4T1 mouse breast cancer cells both *in vitro* and *in vivo* and observed a significant dose-dependent decrease in VEGF expression in cells treated with fucoidan. Further, in a mouse breast cancer model using 4T1 cells, intraperitoneal injections of 10 mg/kg body weight fucoidan from *F. vesiculosus* for 20 days markedly reduced the number of microvessels. Using immunohistochemistry, fucoidan was shown to reduce VEGF expression compared to the control group [33]. In contrast, Zhu *et al.* reported that fucoidan did not suppress angiogenesis and VEGF expression in human hepatocarcinoma cell lines treated with 10 to 200 µg/mL of a commercial fucoidan purified from *Sargassum* spp. Similarly no changes in VEGF expression were observed in xenograft tumors developed in nude mice following 20 to 200 mg/kg/body weight fucoidan injected intraperitoneally once a day over 25 days [34]. It is postulated that different effects are observed with fucoidans of various MWs and molecular structures and this is reviewed by Kwak [13].

#### 3.1.4. Fucoidan and Metastasis

In 1987, Coombe *et al.* demonstrated that fucoidan significantly decreased tumor cells metastasis to the lungs in animals that were intravenously injected with rat mammary adenocarcinoma 13762 MAT cells [35]. It was first reported that fucoidan inhibits cell invasion through competing with tumor cell binding with laminin in the basement membrane [36]. Subsequent studies then revealed that fucoidan binds to fibronectin with high affinity and prevent attachment of tumor cells. In agreement with this study, fucoidan reduced the spread of human breast adenocarcinoma cells plated on a surface containing fibronectin [37].

Selectin inhibition by fucoidan interferes with tumor cell–platelet interaction. In Cumashi *et al.* study [38], highly metastatic MDA-MB-231 breast cancer cells were plated in platelet-coated plates in the presence or absence of 100 µg/mL fucoidan. The number of cells attached to the platelets decreased by 80% in the presence of fucoidan. Interaction of tumor cells with platelets is one of the key factors in facilitating the early steps of tumor cell migration. During tumor cell migration, most circulating tumor cells do not survive attack from immune cells or the shear forces of the blood stream. However, they can attach to platelets to induce platelet aggregation allowing the tumor cell cluster to survive in the micro-vascular system. It was concluded that fucoidan inhibited P-selectin residing on the platelet surface and led to reduced number of attached tumor cells. Fucoidan can also inhibit other adhesion molecules such as integrins residing on the tumor cell surface and can modify distribution of their subunits.

Tumor invasion requires the secretion of proteolytic enzymes by tumor cells to break down the extracellular matrix (ECM) proteins (e.g., collagen, fibronectin and laminin), with the matrix metalloproteinases (MMPs) MMP-2 and MMP-9 playing a major role. Fucoidan attenuates both expression and activity of these enzymes [39].

#### 3.1.5. Fucoidan and Signaling Pathways

The extracellular signal-regulated kinase (ERK) pathway (or Ras/Raf/MAPK pathway) is often hyperphosphorylated and upregulated in a variety of human cancers. The potential for developing anticancer agents that cause ERK’s dephosphorylation and pathway blockade have been explored. Various studies have shown that fucoidan inhibits tumor cell proliferation by decreasing ERKs activity through reduction of its phosphorylation [15,40] while several studies have proposed that fucoidan causes ERK activation rather than inactivation [41,42]. To explain these contradictions, it should be noted that the ERK signaling pathway is highly complex. It induces a range of different responses including cell proliferation, differentiation, migration and apoptosis depending on cell type, the type of stimulus and duration of activation [43]. Therefore, some of the contradictory results of the aforementioned studies can be explained by different fucoidan extracts with different molecular structures being used on different tumor cell types. Another complication is that different studies have examined ERK phosphorylation over different time periods ranging from 10 min to 48 h. Jin *et al.* reported increased ERK1/2 phosphorylation in HL-60 leukemic cell line 10–15 min after fucoidan treatment. The phosphorylation returned to the basal level after 1 h [23]. In Lee *et al.* study, crude fucoidan progressively diminished phosphorylation of ERK1/2 from 1 h to 9 h after treatment [39].

JNK and p38 are other MAPK superfamily members whose activity is altered by fucoidan. Fucoidan induced cell death in breast cancer cells through phosphorylation and activation of JNK and p38 after 30 min. The fucoidan-induced apoptosis significantly annulled in the presence of JNK inhibitor, indicating critical role of JNK in fucoidan-mediated apoptosis [14].

**Figure 1:**
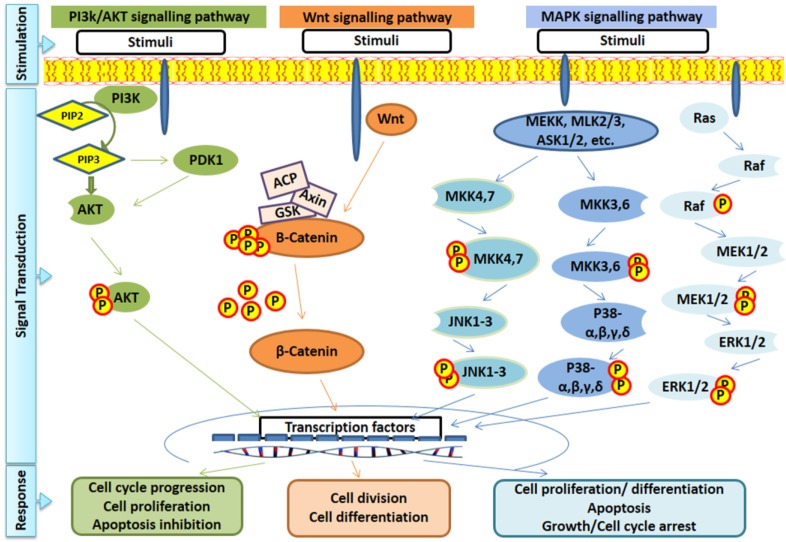
Overview of main signal transduction pathways involved in cell proliferation and apoptosis.

Similarly, the PI3K/AKT, GSK and Wnt pathways have been shown to be triggered by fucoidan. PI3K/AKT pathway generally inhibits apoptosis. AKT over-activation is also associated with drug resistance and tumor cell survival. As a result, deactivating this pathway could be another potential target for anti-cancer drug development. Most of the studies have reported inactivation of AKT by fucoidan. PI3k, an upstream molecule of AKT, is also inhibited by fucoidan [39]. Upregulation of the Wnt signaling pathway is believed to have a critical role in prostate cancer development, survival and progression. Fucoidan from *F. vesiculosus* activated GSK-3β in PC3 human prostate cancer cells resulting in hypo-phosphorylation and inactivation of β-catenin, a critical component of the Wnt pathway (Figure 1) [18]. Figure 1 represents an overview of the mentioned signaling pathways.

#### 3.1.6. Fucoidan and the Immune System

The effects of fucoidan on molecules of the immune system have been studied both *in vitro* and *in vivo* and effects on both cellular and humoral elements have been described. Fucoidan increases both activity and number of natural killer (NK) cells *in vivo* [44,45]. Increase in the number of cytotoxic T-cells (CTLs) has also been reported. A high-molecular-weight (HMW) fucoidan from *Cladosiphon okamuranus* (200–300 kDa) induced a large increase in the proportion of murine cytotoxic T cells [46]. Investigation of the role of fucoidan on dendritic cell (DC)-mediated T-cell cytotoxicity has revealed that the stimulation of CTLs was more effective in fucoidan-treated DCs as CTLs co-cultured with fucoidan-treated DCs exerted a high level of specific lysis of breast cancer cells [47].

In a recent study, the role of fucoidan in DCs function and its adjuvant effect have been examined *in vivo*. Fucoidan was systemically administrated to mice by intraperitoneal injection. Examination of the spleen DCs revealed up-regulation of maturation markers as well as production of IL-6, IL-12 and TNF-α. Fucoidan was then used as an adjuvant *in vivo* with ovalbumin antigen and induced Th1 mediated immune response and CTL activation [48]. 

#### 3.1.7. Fucoidan and Malignant Transformation *in Vitro* and *in Vivo*

Few studies have reported the potential of fucoidan to inhibit neoplastic transformation. Teas *et al.* fed rats with dietary seaweed (*Laminira*) for 55 days and administrated the carcinogen 7,12-dimethylbenz(a) anthracene intragastrically. Following 26 weeks monitoring, experimental rats showed a significant delay in the median time for tumor appearance (19 *vs.* 11 weeks in the control group) [49].

Transforming growth factor β1 (TGFβ1) is believed to promote tumor development and metastasis through epithelial to mesenchymal transition (EMT), a process that enables epithelial cells migrate to distant areas during late stages of breast cancer development [50]. To trigger tumor progression, TGFβ1 recruits TGF receptors (TGFR) residing on the cell surface. The investigations of effects of fucoidan on TGFβ1-promoted carcinogenesis in MDA-MB-231 breast cancer cells have indicated that fucoidan decreased the expression of TGFRs and affected the downstream signaling molecules, which are involved in TGFβ1-mediated EMT [41]. 

Epidermal growth factor (EGF) is another carcinogenesis promoter, which induces tumor transformation through overexpression and activation of EGF receptor (EGFR). EGFR has a key role in cell proliferation and differentiation and many carcinomas arise from its mutations [51]. Lee *et al.* examined the role of fucoidan on the activation of EGFR and EGF-mediated neoplastic transformation [52]. They utilized murine JB6 Cl41 epidermal cells and induced cell transformation by EGF in the presence of fucoidan from *L. guryanovae*. Fucoidan markedly reduced the EGFR activation through hypo-phosphorylation. It also inhibited EGF-tumorigenic activity through inhibition of AP-1, a transcription factor responsible for cell proliferation regulation.

### 3.2. Fucoidan Metabolism

Fucoidanase, the enzyme responsible for fucoidan hydrolysis, has only been found in brown seaweed and marine microorganisms such as some marine bacteria and fungi [53] and not in humans. It is possible that the acidic conditions in the stomach could degrade fucoidan, but it has been reported that the low gastric pH does have restricted effects on fucoidan [54].

Small amounts of dietary fucoidan can be endocytosed and cross the intestinal wall directly without breaking down [54]. In Tokita *et al.* study, 10 volunteers were given oral fucoidan and the concentrations of fucoidan in the serum and urine were analyzed. Fucoidan was detectable 3 h after administration and increased to 100 ng/mL in serum and 1000 ng/mL in urine. However the rate of absorption in the small intestine was highly variable among the participants. The MW of fucoidan in serum was similar to administered fucoidan indicating that fucoidan was not hydrolyzed by digestive enzymes [55]. However, the MW of the fucoidan detected in urine was significantly smaller than the ingested fucoidan suggesting that fucoidan is degraded in the excretory system and possibly the kidney and not by intestinal enzymes or normal flora.

To evaluate the fucoidan uptake process by cells, the internalization of LMW fucoidan into rabbit smooth muscle cells (SMCs) was analyzed. Fucoidan was shown to be internalized by endocytosis at 6 h. The number of vesicles containing fucoidan increased in the peri-nuclear region at 24 h, but nuclear internalization was not observed at any time during the study [56]. However, examining the transport of a native fucoidan from *Cladosiphon okamuranus* with MW of 80 kDa revealed a poor permeation of fucoidan across the human colon adenocarcinoma Caco-2 cell monolayer [57].

Regarding the specific ligands by which fucoidan binds to the cells surface, several molecules have been implicated including class A macrophage scavenger receptors for fucoidan attachment to macrophages [58] as well as adhesion molecules such as L-selectin and P-selectin [59] and integrins [60]. However, some reports have shown fucoidan mediates apoptosis through selectin-independent mechanisms [15].

### 3.3. Fucoidan as a Synergistic Anti-Cancer Agent

The ability of fucoidan to synergize with standard anti-cancer agents and/or reduce toxicity has recently been investigated. Ikeguchi *et al.* examined the synergistic effect of a HMW fucoidan with colorectal cancer chemotherapy agents; oxaliplatin plus 5-fluorouracil/leucovorin (FOLFOX) or irinotecan plus 5-fluorouracil/leucovorin (FOLFIRI). The test patients received 150 mL/day for 6 months of liquid that contained 4.05 g fucoidan. From the commencement of chemotherapy, toxicities and chemotherapy efficiency were compared. Fucoidan showed no side effects such as allergic dermatitis. Diarrhea, neurotoxicity and myelosuppression were not suppressed by fucoidan, whereas general fatigue was significantly decreased from 60% to 10%. The patients were followed for approximately 15 months and the survival rate of the patients who received fucoidan was longer than that of the control participants; however the difference was not significant, probably due to the small numbers [61]. 

Fucoidan affects the migration and invasion of multiple myeloma (MM) cells treated with chemotherapy drug cytarabine. The human myeloma cell lines RPMI8226 and U266 were treated with crude fucoidan from *F. vesiculosus* for 72 h and then cytarabine for 6 h. Fucoidan reduced cell migration through a Boyden chamber and down-regulated expression of CXCR4 and MMP-9 [62]. Fucoidan from *Saccharina cichorioides* has been reported to synergize with the anti-tumor activity of low dose resveratrol (a natural polyphenol extracted from foods and beverages) on invasive and highly motile HCT 116 colon cancer cell line [63]. In the colony formation assay, fucoidan plus resveratrol reduced the colony number by 60% compared to 34% and 27% in resveratrol alone or fucoidan alone, respectively. 

Zhang *et al.* studied the combinatory effect of fucoidan and three commonly used anti-cancer agents; cis-platin (CDDP), tamoxifen (TAM) and paclitaxel (Taxol) on signal transduction pathways. Fucoidan from *Cladosiphon navae-caledoniae* plus anti-cancer agents reduced the ERK phosphorylation in MDA-MB-231 breast cancer cells compared to untreated control or fucoidan alone [64]. Dietary fucoidan synergistically reduced cell growth in the OE33 cell line when it was combined with lapatinib, a targeted therapy that acts as a tyrosine kinase inhibitor in advanced HER2-positive breast cancer cells [65]. 

In a xenograft transplantation study, the effect of fucoidan alone or in combination with cyclophosphamide was examined on tumor growth. Nine days after the injection of Lewis lung carcinoma cells into mice, fucoidan from *Fucus evanescens* was administered to animals alone or combined with cyclophosphamide. The fucoidan group showed marked antitumor (33% tumor growth inhibition) and anti-metastatic (29% reduction of the number of metastases) activities. However, fucoidan did not exhibit a synergistic effect with cyclophosphamide on tumor growth, but significantly decreased the lung cancer cells metastasis [66].

### 3.4. Why Fucoidan Usage is Complicated?

Despite the promising results about the anti-cancer effect of fucoidan, there are still challenges impeding utilization of fucoidan in the clinic. Variable and contradictory results being influenced by endogenous and exogenous factors in fucoidan usage are of the main concerns. In this section we will summarize important conditions, which have been undertaken in different experiments and have led to such variable results in reported studies.

#### 3.4.1. Structure and Molecular Weight Variation

Fucoidan is composed of α-(1-2) or α-(1-3)-linked L-fucose with a fucose content of 34-44%. It also contains various amounts of other monosaccharaides such as galactose, mannose, xylose and uronic acid all of which make up less than 10% of the total fucoidan structure [67,68]. The sulfate groups in fucoidan structure are mainly at position 4 but they can also occupy position C_2_ and occasionally C_3_ [53]. The fucoidan structure and monosaccharide composition vary depending on different factors such as the source of fucoidan, the time and location of harvesting and the extraction method, which can affect the fucoidan’s bioactivities. Most anti-cancer studies of fucoidan have used a commercially available crude fucoidan extracted from *Fucus vesiculosus* (Sigma Co. St. Louis, MO, USA). Some groups have extracted and purified fucoidan in their own laboratories. *Okinawa mozuku*, *C. Okamuranus* tokida, *Sargassum* sp. and *Undaria pinnatifida* are the most common fucoidans examined in cancer studies. 

Cumashi *et al.* studied different biological aspects of fucoidan from nine different species of brown seaweed in rats [38]. Analysis of P-selectin-mediated neutrophil adhesion to platelets revealed that extracted fucoidans from only some sources like *F. evanescens* and *A. nodosum* could serve as more efficient P-selectin inhibitors. Furthermore, in contrast to other sources, fucoidan from *C. okamuranus* did not exert anti-coagulant activity, which was suggested to be due to high content of 2-O-a-d-glucuronyl substituent in the polysaccharide chain of fucoidan from *C. okamuranus*.

Sulfation is another key factor in fucoidan bioactivity. More sulfation is linked with greater bioactivity and thus researchers have produced over-sulfated fucoidans to enhance its biological properties [36]. It has been suggested that over-sulfation causes higher negative charge in the molecule which can facilitate formation of fucoidan-protein complexes involved in cell proliferation [69]. 

Molecular weight is another crucial factor in fucoidan activity. Cho *et al.* produced three fucoidan fractions with molecular weights of <5, 5–30 and >30 kDa and reported that the F_5-30K_ showed the most tumor growth inhibitory effect despite the sulfate amount in F_<5K_ being greater than in the two other fractions [70]. 

The extraction method can also affect fucoidan’s bio-properties. Fucoidan from *Undaria pinnatifida* was hydrolyzed using different hydrolysis conditions and their anti-cancer activity was compared *in vitro*. The native fucoidan showed 37% anti-cancer activity; hydrolyzed fucoidan generated under mild conditions (in boiling water with HCl for 5 min) exhibited 75.9% anti-tumor activity; whereas hydrolyzed fucoidan generated under harsh conditions (microwave for more than 90 s) slightly enhanced the anti-cancer effect [71].

#### 3.4.2. Fucoidan Dose and Route of Administration

As fucoidan is a large highly branched molecule, the dosage for *in vitro* studies mostly resides in the range of µg/mL and not ng/mL. However, there is a large variation in the doses. Vischchuk *et al.* treated HCT-116 colon cancer cells with 100–800 μg/mL fucoidan from the brown alga *Saccharina cichorioides* Miyabe and observed that fucoidan exerted a low cytotoxicity and there was less than 15% reduction in cell number with the high dose of 800 μg/ml after 24 h [63]. In contrast, Kim *et al.* demonstrated that 20 μg/mL fucoidan from *F. vesiculosus* caused 37% growth inhibition in the same cell line after 72 h [24]. Though the difference between incubation times (24 h *vs.* 72 h) should be considered, the dose difference (800 μg/mL *vs.* 20 μg/mL) was substantial. The source of fucoidan appears to be the main factor leading to variation in results. Though most researchers have utilized dosages of less than 1 mg/mL, there are reports of use of up to 3 mg/mL fucoidan.

Regarding the *in vivo* studies, both dose and the route of administration can affect outcome. To select the most effective dose, mice were treated with various doses of fucoidan (10–400 mg/kg body weight) followed by total-body irradiation. The mice injected with 100 mg/kg body weight fucoidan showed the best survival rate at 30 days post-irradiation [72]. Other studies have used various doses ranging from 5 mg/kg to 100 mg/kg and occasionally doses up to 500 mg/kg/body weight of different fucoidan extracts. The amount and number of doses of fucoidan administration has also been shown to be important for *in vivo* studies. Alekseyenko *et al.* studied mice with lung carcinoma that were treated with fucoidan from *Fucus evanescence*. They found that a single injection of 25 mg/kg/body weight of fucoidan did not inhibit tumor cell proliferation, while three-time injections of 10 mg/kg/body weight significantly reduced tumor growth and metastasis [66]. Most *in vivo* studies of anti-tumor activity have selected intraperitoneal (IP) injections, but subcutaneous (SC) or intravenous (IV) routes of administration have also been used. Oral fucoidan is another route for *in vivo* delivery either for its anti-tumor properties following tumor induction or as a neoplastic transformation inhibitor administered prior to cancer induction. Taken together, these studies indicate that different delivery routes can affect the fucoidan metabolism *in vivo* and lead to variable outcomes.

### 3.5. Fucoidan Toxicity

Whilst fucoidan consumed in food in the form of 4% of the total dry weight of brown seaweeds is generally regarded as safe, the fucoidan used for research is a highly purified extract. For *in vitro* studies, researchers have utilized normal cells such as normal fibroblasts alongside tumor cell lines and reported that fucoidan did not induce apoptosis within normal cells at the doses which were toxic for cancer cell lines. A very high dose of 3 mg/mL fucoidan suppressed the viability of peripheral blood mononuclear cells from healthy donors by 25% compared to 60%–90% in five different leukemic T-cells [20]. *In vivo*, oral administration of up to 1 g/mL/body weight *Undaria pinnatifida* fucoidan was non-toxic in mice but higher doses (2 g/mL/body weight) induced changes in thyroid weight and altered levels of triglyceride and alanine transaminase activity [73]. In another study, daily administration of 300 mg/kg/body weight fucoidan from *Laminaria japonica* in Wister rats over 6 months did not induce any adverse side effects, but higher doses (900–2500 mg/mL) resulted in coagulopathy and markedly elevated clotting time [74].

Toxicity has also been examined in the context of fucoidan use as adjuvant. Oh *et al.* examined the combinatory effect of fucoidan with the standard anti-Her2 inhibitor lapatinib in different breast cancer cell lines *in vitro* [65] and found that fucoidan decreased the efficiency of lapitinib and exerted antagonistic effects on cell proliferation in a few cell lines. Examining the effect of combination of fucoidan from *Fucus evanescence* with cyclophosphamide, 7 out of 10 mice that were injected with 25 mg/kg/body weight fucoidan plus cyclophosphamide died whereas of the mice that were treated with fucoidan alone, 3 out of 10 died [66].

Fucoidan has been examined in several clinical trials mainly for its anti-coagulant and anti-viral properties. Administration of capsules containing 560 mg fucoidan from *Undaria pinnatifida* for up to 24 months did not induce any side effect when the participants took 4 capsules a day [75]. In Mori *et al.* [76] and Irhimeh *et al.* [77] studies, daily consumption of 5 capsules contained 166 mg fucoidan from *C okamuranus* Tokida for over one year and 3 g HMW fucoidan from *Undaria pinnatifida* for up to 12 days, respectively, were revealed to be safe. However, Irhimeh *et al.* demonstrated that orally administered fucoidan affected coagulation tests and prolonged the aPTT, thrombin time and AT-III. Other studies have also shown the potential of bleeding complication development due to fucoidan’s anti-thrombotic property [78]. Diarrhea is another reported side effect, which was seen in 4 out of 17 participants within 1 month of daily administration of 6 g fucoidan [79].

When a blend of three different extracts (from *Fucus vesiculosis* (85% w/w), *Macrocystis pyrifera* (10% w/w), and *Laminaria japonica* (5% w/w)) in capsules containing up to 187.5 mg were daily given to volunteers, a statistically significant change in the potassium level was seen after 28 days. Although, the change was minor and within the clinical reference range [80].

## 4. Conclusions

The goal of cancer treatment is eradication of tumor cells ideally with minimal damage to healthy tissues. Because of the side-effects of many current treatments, the use of natural substances of low toxicity is of interest. A number of *in vitro* and *in vivo* studies have indicated that fucoidan contains strong anti-cancer bioactivity. Since fucoidan also possesses immunomodulatory effects, it is postulated that it may have protective effects against development of side effects when it is co-administered with chemotherapeutic agents and radiation. 

In this report, we reviewed the underlying cellular mechanisms by which fucoidan induces cell death within tumor cells and increases the survival rate of tumor-bearing animal models by suppression of metastasis and angiogenesis. However despite numerous promising pre-clinical reports, there are few reported clinical studies so far [61]. In this review we also discussed the challenges impeding utilization of fucoidan in the clinic which include the complex heterogeneous structure of fucoidan, highly variable doses, different administration routes and possible negative interactions with chemotherapy. Due to the wide variation of fucoidan structure and to make future experiments reproducible, it is recommended that the critical bioactivity factors such as fucoidan content, sulfate content, monosaccharide constituents and molecular weight be reported. Attention to these factors will be likely to lead to more consistent reports and ultimately produce the required evidence to underpin clinical studies in near future.

## Authors Contribution

FA conducted the literature research and drafted the manuscript. RML carried out the supervision and edited the manuscript. GMW carried out the supervision and edited the manuscript. AFH carried out the supervision and edited the manuscript. JLD carried out the supervision and edited the manuscript.
